# 3DIV update for 2021: a comprehensive resource of 3D genome and 3D cancer genome

**DOI:** 10.1093/nar/gkaa1078

**Published:** 2020-11-27

**Authors:** Kyukwang Kim, Insu Jang, Mooyoung Kim, Jinhyuk Choi, Min-Seo Kim, Byungwook Lee, Inkyung Jung

**Affiliations:** Department of Biological Sciences, Korea Advanced Institute of Science and Technology (KAIST), Daejeon 34141, Korea; Korean Bioinformation Center, Korea Research Institute of Bioscience and Biotechnology (KRIBB), Daejeon 34141, Korea; Department of Biological Sciences, Korea Advanced Institute of Science and Technology (KAIST), Daejeon 34141, Korea; Korean Bioinformation Center, Korea Research Institute of Bioscience and Biotechnology (KRIBB), Daejeon 34141, Korea; Korean Bioinformation Center, Korea Research Institute of Bioscience and Biotechnology (KRIBB), Daejeon 34141, Korea; Korean Bioinformation Center, Korea Research Institute of Bioscience and Biotechnology (KRIBB), Daejeon 34141, Korea; Department of Biological Sciences, Korea Advanced Institute of Science and Technology (KAIST), Daejeon 34141, Korea

## Abstract

Three-dimensional (3D) genome organization is tightly coupled with gene regulation in various biological processes and diseases. In cancer, various types of large-scale genomic rearrangements can disrupt the 3D genome, leading to oncogenic gene expression. However, unraveling the pathogenicity of the 3D cancer genome remains a challenge since closer examinations have been greatly limited due to the lack of appropriate tools specialized for disorganized higher-order chromatin structure. Here, we updated a 3D-genome Interaction Viewer and database named 3DIV by uniformly processing ∼230 billion raw Hi-C reads to expand our contents to the 3D cancer genome. The updates of 3DIV are listed as follows: (i) the collection of 401 samples including 220 cancer cell line/tumor Hi-C data, 153 normal cell line/tissue Hi-C data, and 28 promoter capture Hi-C data, (ii) the live interactive manipulation of the 3D cancer genome to simulate the impact of structural variations and (iii) the reconstruction of Hi-C contact maps by user-defined chromosome order to investigate the 3D genome of the complex genomic rearrangement. In summary, the updated 3DIV will be the most comprehensive resource to explore the gene regulatory effects of both the normal and cancer 3D genome. ‘3DIV’ is freely available at http://3div.kr.

## INTRODUCTION

Hi-C (high-throughput chromatin conformation capture), which captures genome-wide all-to-all chromatin contacts in an unbiased manner, enables visualization of the genome organization in three-dimensional (3D) nuclear space in the form of a Hi-C contact map (1). Analysis of Hi-C contact maps has successfully unveiled various properties of 3D chromatin structure, such as multilayered genome organization (1–3), dynamic reorganization during various biological processes (4–6), regulation of gene expression (7,8), and disruption in diseases (9–11).

Recent studies have revealed that genomic rearrangements in the cancer genome often disorganize higher-order chromatin structures, which can be pathogenic (12). For example, structural variations (deletions, inversions, duplications, and translocations) disrupting topologically associating domains (TADs) cause a failure of proper interactions between the promoter and *cis*-regulatory elements, leading to oncogenic gene expression (13–15). Thus, interpretation of the 3D cancer genome can provide additional insights in understanding aberrant gene regulatory mechanisms in cancer.

Despite the merits of utilizing Hi-C, integrative analysis has been greatly limited by the difficulties in processing large-scale Hi-C contact maps and visualizing the 3D cancer genome through mapping one-dimensional genomic rearrangements to 3D space. The frequently observed complex genomic rearrangements such as chromoplexy (16,17) further exacerbate these limitations, requiring novel visualization tools for extensively rewired chromatin contacts.

To resolve these issues, we updated 3DIV (18) and developed new browsing tools specializing in the 3D cancer genome. One hundred and sixty-eight cancer cell line/tumor and 52 unpublished colorectal cancer Hi-C datasets were added together with the lists of sample-specific or pan-cancer defined WGS structural variations (SVs). We also developed unique live manipulation and visualization tools for the disorganized 3D cancer genome. These unique features enable users to perform multiple tasks, such as examining the impact of SVs on the 3D genome, configuring rearranged 3D chromatin structure, or simulating chromatin contacts of highly rearranged genomes under user-specified order. In summary, compared to the other existing databases, the updated 3DIV provides the largest number of Hi-C samples and covers most of the required functionality in navigating the 3D cancer genome (Table 1).

**Table 1.** Comparison of the updated 3DIV and other 3D genome databases as of October 2020

Software	Number of samples^a^	Hi-C contact map	TAD annotation	One-to-all interaction	Interaction table	Distance normalization	Interactive Hi-C contact map browsing	Live manipulation of genomic rearrangement	Structural variation annotation	Data type
3DIV 2021 Update	401	✓	✓	✓	✓	✓	✓	✓	✓	Hi-C and capture Hi-C
3DIV	80	✓	✓	✓	✓	✓	✓			Hi-C
4D Nucleome	337^b^	✓					✓			Hi-C, capture Hi-C, ChIA-PET, and Hi-C variants
Nucleome Browser	138^c^	✓					✓			Hi-C and Hi-C variants
WashU Epigenome Browser	36^c,d^	✓					✓			Hi-C, capture Hi-C, and ChIA-PET
HiView	2		✓	✓	✓	✓	✓			Hi-C
HUGIn2	83	✓	✓	✓	✓	✓				Hi-C, capture Hi-C, and HiChIP
3D Genome Browser	113	✓	✓							Hi-C, ChIA-PET, capture Hi-C
GITAR	20^c^	✓	✓							Hi-C
Hi-C Data Browser	69	✓		✓						Hi-C, capture Hi-C, and ChIA-PET
3Disease Browser	6	✓	✓						✓	Hi-C
ChromContact	6			✓						Hi-C
ENCODE	95^e^									Hi-C and ChiA-PET

^a^Only Hi-C and its variant experiments in ‘Data type’ column conducted on the human samples were considered. Multiple replicates were counted as one.

^b^4D Nucleome uses HiGlass display.

^c^Nucleome Browser, GITAR, and WashU Epigenome Browser can visualize user-uploaded data.

^d^Samples uploaded as hg38 were counted.

^e^In the case of ENCODE, the data provided is different according to the uploaded processing results. Part of the ENCODE data has been processed to be used as an input file for Juicebox.

## MATERIALS AND METHODS

### Collection of Hi-C, epigenetic feature, and structural variation data

We collected all Hi-C results published after July 2017 from the GEO (Gene Expression Omnibus) database. Preexisting Hi-C samples published before July 2017 in the 3DIV database were also reprocessed for updates. As a result, we processed ∼230 billion raw reads covering 220 cancer Hi-C samples for 18 cancer-types, 153 normal Hi-C samples, and 28 promoter capture Hi-C samples. Details of the processed samples are listed in Supplementary Table S1.

For each normal Hi-C sample, 3DIV provides GWAS-SNPs, histone ChIP-seq signals, and super-enhancers for the functional characterization of the interacting genomic regions. In total, we collected 310 histone ChIP-seq results, including H3K27ac, H3K27me3, H3K4me1, H3K4me3, H3K9ac, and CTCF from the matched or most relevant cell/tissue-types. We uniformly processed these large epigenome datasets to provide ChIP-seq enrichment signals for each 5 kb genomic bin (Supplementary Table S2 and see Supplementary Methods). Super-enhancer annotations were also obtained from dbSUPER (19). In addition, disease-associated single nucleotide polymorphism (SNP) information was obtained from the GWAS catalog (20) (see Supplementary Methods).

In the case of ‘cancer Hi-C’ data, 3DIV provides a list of large-scale genomic rearrangements within a selected genomic region. Coordinates, SV type, and read direction of the whole-genome sequencing (WGS)-identified structural variations were provided. If available, SV data obtained from the matched WGS data were provided. If not, public pan-cancer SV data corresponding to each sample's cancer-type were provided. The pan-cancer SV data were obtained from the International Cancer Genome Consortium (ICGC), which includes data from The Cancer Genome Atlas (TCGA) (21) and the Pan-Cancer Analysis of Whole Genomes (PCAWG) consortium (22). Additional information is available in the Supplementary Methods.

### Processing of collected Hi-C data

FASTQ files of each Hi-C sample were aligned to the hg38 reference genome, filtered, and normalized by covNorm (23) (see Supplementary Methods). The processing procedures depend on the sample type. In the case of cancer cell line/tumor tissue samples and their control samples marked as ‘cancer Hi-C’, both *cis-* and *trans-* interactions were prepared at a 40kb resolution. For normal Hi-C samples, *cis*-only interactions within a 2Mb distance were processed at a 5kb resolution. For normal Hi-C data, topologically associating domain (TAD) annotation was also provided by using TopDom (24) and DomainCaller (2) with two different window size options (w = 5 and 20 for TopDom and DI calculation = 500kb and 2Mb for DomainCaller) (see Supplementary Methods).

### Implementation of the updated 3DIV

Similar to the previous version of 3DIV, a three-layered architecture consisting of data (backend), logic (middleware), and presentation tiers (frontend) was used. Unlike the previous version, HTML5 functions were used instead of D3.js functions to rapidly display and rearrange large *trans* Hi-C contact maps of cancer samples. The frontend was optimized in the Google Chrome and Safari environments. The Java Spring Framework and MyBatis Framework were used to implement middleware that connects the frontend and MySQL-based backend (Supplementary Figure S1).

## RESULTS

### Contents update for 3DIV 2021

**Figure 1.**
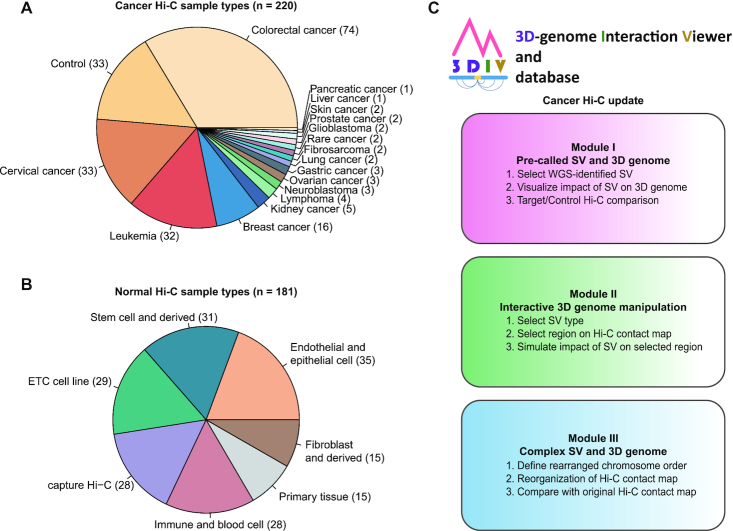
Overview of the 3DIV update. (**A**) Pie chart presenting the number of samples per cancer-type covered by newly added cancer Hi-C samples. Control indicates Hi-C data used as controls for cancer samples including normal cell and tissue. (**B**) Pie chart showing the number of samples per cell/tissue-type in the updated normal Hi-C database. (**C**) Three user-accessible main modules and their functions in the updated 3DIV.

As part of a major update in 3DIV 2021, 3DIV contents have been expanded to the 3D cancer genome. To this end, we processed 220 cancer Hi-C samples for 18 cancer-types, including 159 cell line and 61 blood/tumor samples (Figure 1A). The Hi-C experiments conducted with various treatments such as drugs, CRISPR/Cas9, or gene knockout with cancer cell lines were also included, which allows investigating 3D chromatin structure dynamics under multiple conditions. The ‘Control’ class contains normal samples for the corresponding cancer type, such as primary blood cells or normal colon tissues.

Though the main content of the update is 3D cancer genome, renewal and expansion of the preexisting 3DIV Hi-C database were also performed. A total of 153 normal Hi-C samples is now available in the database by processing approximately 116 billion raw read counts, which identified 31 billion valid chromatin interactions (Figure 1B). Normal Hi-C samples were classified into 6 types, comprising Hi-C data originated from the stem cell and derived, immune and blood cell, primary tissue, fibroblast and derived, endothelial and epithelial cell, etc. cell lines. Similar to the cancer Hi-C database, Hi-C results obtained under various experimental conditions were also added.

Various derivative forms of Hi-C protocols such as capture Hi-C (25) or HiChIP (26) were developed to further examine the role of 3D chromatin structures in gene regulation. As a follow-up, promoter capture Hi-C data generated from 28 different human cell/tissue-types (23) were added with own visualization functions (Supplementary Figure S2). Long-range significant promoter-centered interactions can be visualized and compared across multiple human cell/tissue-types.

Due to the collection of hundreds of Hi-C samples, we implemented a sequential sample categorization scheme, enabling users to easily navigate Hi-C samples of interest. Samples to be visualized can be quickly selected by querying samples’ keywords in the search window or by sequentially selecting sample characteristics such as types (e.g., cancer-, cell- or tissue-type), sample property (e.g., cell line name), experimental conditions (e.g., drug-treated), and sample name. Statistics of updated samples and download-ready data are also available at the 3DIV website.

### Development of cancer Hi-C analysis and visualization modules

Unlike normal samples, large-scale SVs such as deletions, duplications, inversions, and translocations are frequently observed in the cancer genome, which are part of the main factors that lead to changes in the 3D chromatin structure. Distal genomic regions can form new spatial chromatin contacts by large-scale SVs, which generates highly visible SV break-end-enriched contact reads on Hi-C contact maps (27). Using this feature, a Hi-C contact map can be utilized to intuitively interpret the effects of large-scale SVs on genome organization and compensate for the limitations of WGS in detecting large-scale SVs (28). In this regard, three modules were developed for the 3DIV update: visualization with WGS-identified SVs, manipulation of Hi-C contact maps by clicking, and recombination of Hi-C contact maps based on user-defined chromosome order (Figure 1C). Explanation figures and working examples are available for each module page with detailed tutorials. Descriptions of the modules will be covered in detail below.

#### Module I. Visualization of the effect of WGS-identified structural variations on the 3D genome

**Figure 2.**
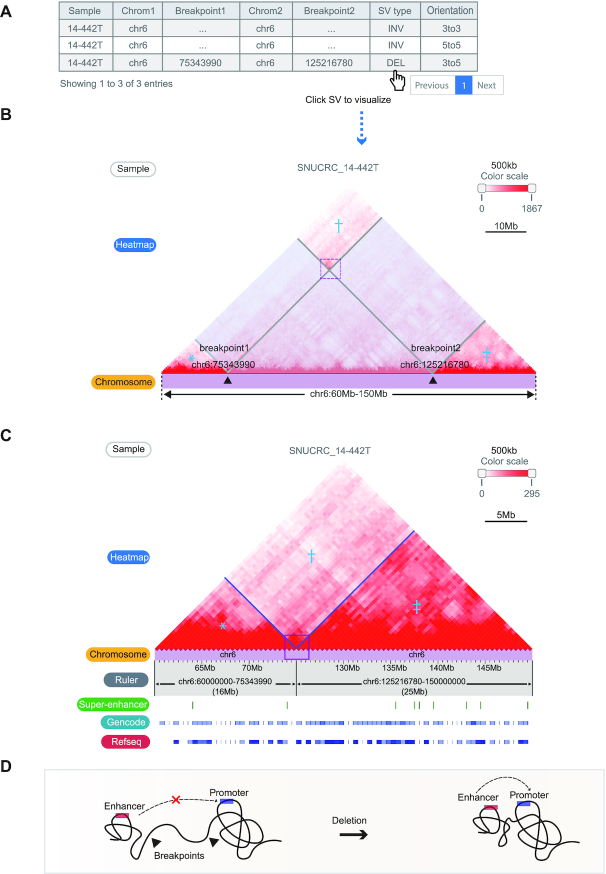
Visualizing effect of the structural variations on the Hi-C contact map. (**A**) The table of WGS-identified structural variations from sample ‘SNUCRC_14–442T’ within the chr16:60–150 Mb region. (**B**) Affected regions (translucent boxes) on the Hi-C contact map by the SV ‘chr6 75343990 chr6 125216780 DEL 3to5’ can be visualized by clicking the SV in the table. The regions marked with an asterisk (*), dagger (†), and double dagger (‡) are reorganized by an exemplified deletion event. (**C**) Reorganized Hi-C contact maps (*, †, and ‡ in Figure 2B) showing breakpoint-crossing Hi-C signals (purple line box). Gene density and super-enhancers at recombined regions are also displayed. (**D**) Illustration showing the interpretation of SV-mediated newly established chromatin contacts in terms of rewired *cis*-regulatory elements and promoters.

The first module receives a genomic region as an input and presents a Hi-C contact map with a list of WGS-identified structural variations of the queried region (Figure 2A). Breakpoint coordinates and types of SVs based on the read direction are also provided. The SV list of the queried sample is presented if available; otherwise, the cancer type-matching SV list obtained from the pan-cancer data is provided.

Clicking the SV on the list first displays the range of the SVs on the Hi-C contact map of the target sample (Figure 2B), and the chromatin contacts changed by the SVs are displayed on the next panel (Figure 2C). The recombination of the Hi-C contact map from Figure 2B to C illustrates a smooth linkage of contact signals crossing breakpoints, indicating the establishment of *de novo* chromatin contacts between the regions proximal to the SV breakpoints. This allows the user to examine the impact of SVs on 3D chromatin contacts and predict their potential regulatory effects by investigating rewired interactions between regulatory elements (Figure 2D).

In addition, the module I is equipped with a function that provides a list of sample/caner-type matched SVs after selecting a sample, which can be used as an input parameter instead of arbitrary genomic coordinates, thereby allowing users to quickly select and examine the region of interest.

#### Module II. Live interactive manipulation of disorganized 3D chromatin structure

WGS-identified SVs can explain a certain portion of changes in Hi-C contact maps, but two methods often show a discrepancy in the detection of SVs (28,29). For example, complex rearrangements and SVs spanning unmappable regions can hinder the formation or detection of the direct breakpoints in WGS. However, spatial contacts between surrounding regions of the breakpoints make these events visible on the Hi-C contact maps. In addition, the function for simulating multiple SVs together is also required to estimate the impact of various genomic rearrangements in the cancer genome.

**Figure 3.**
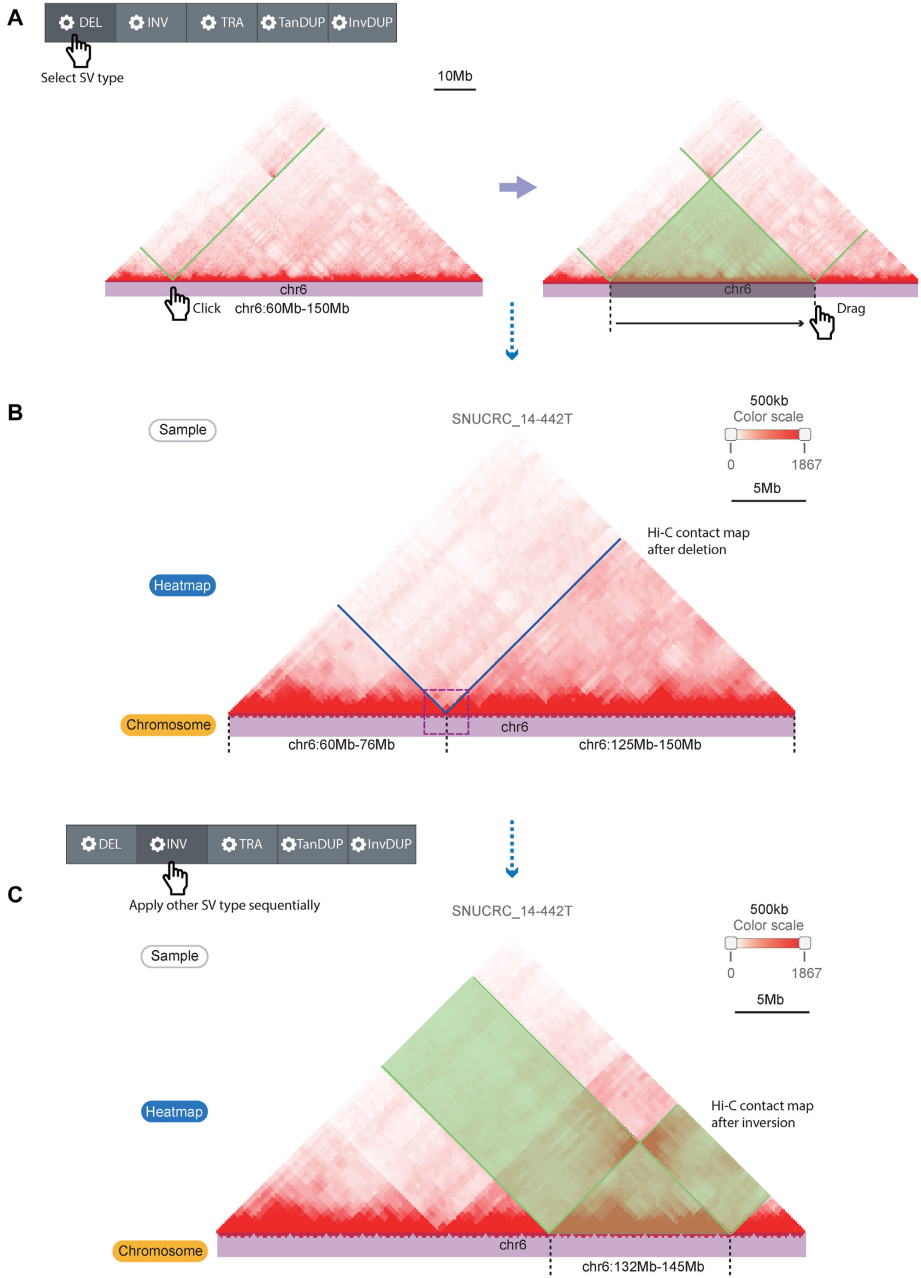
Live Hi-C contact map manipulation function for simulating the effect of SVs. (**A**) Hi-C contact map manipulation by selecting the SV type, clicking one breakpoint, and dragging the cursor on the chromosome track to the next breakpoint. The black dashed lines indicate breakpoint coordinates, and the green line/translucent triangle indicates the affected Hi-C contact map region by the simulated SV type. (**B**) A recombined Hi-C contact map by the simulated SV (deletion) that reproduces the deletion example in Figure 2. (**C**) Applying another SV simulation (inversion, marked with the black dashed lines and translucent green) at the already recombined Hi-C contact map. Translucent green boxes indicate inverted Hi-C signals by the simulation.

To this end, the second module of the updated 3DIV provides live interactive manipulation of a Hi-C contact map. Users can select the region of interest simply by clicking and dragging on a chromosome track (Figure 3A), and the Hi-C contact map will be rearranged based on the SV type that the user selected (Figure 3B). The ruler below the chromosome track provides a detailed scale and a crop function, which shows the sizes of the rearranged fragments and aids users in preparing publication-ready figures from the database. Unlike module I, multiple changes can be sequentially applied to the Hi-C contact map. For example, Figure 3C shows a simulation of an additional inversion after the deletion applied in the Hi-C contact map. The control panel also provides additional manipulations such as adjusting the scale (zooming in or out) or reverting applied changes on the Hi-C contact map. The control panel initially located at the top of the web page follows the user in the form of a sidebar when the web page is scrolled, making it easy to adjust the contact map without scrolling back to the top of the web page. The original contact map marked with changed regions is also provided in parallel with the manipulated contact map, allowing users to compare the altered 3D genome through the simulated SVs, intuitively.

#### Module III. Visualization of the complex SV effect on the 3D genome

Recent studies have identified massively rearranged chromosomes such as chromothripsis and chromoplexy (16,17,27). These complex rearrangements often involve more than dozens of SVs on single or multiple chromosomes, which cannot be intuitively presented on Hi-C contact maps. Additionally, as complex rearrangements often occur as a single catastrophic event, it may be challenging to implement simultaneous rearrangements only by sequential inputs.

**Figure 4.**
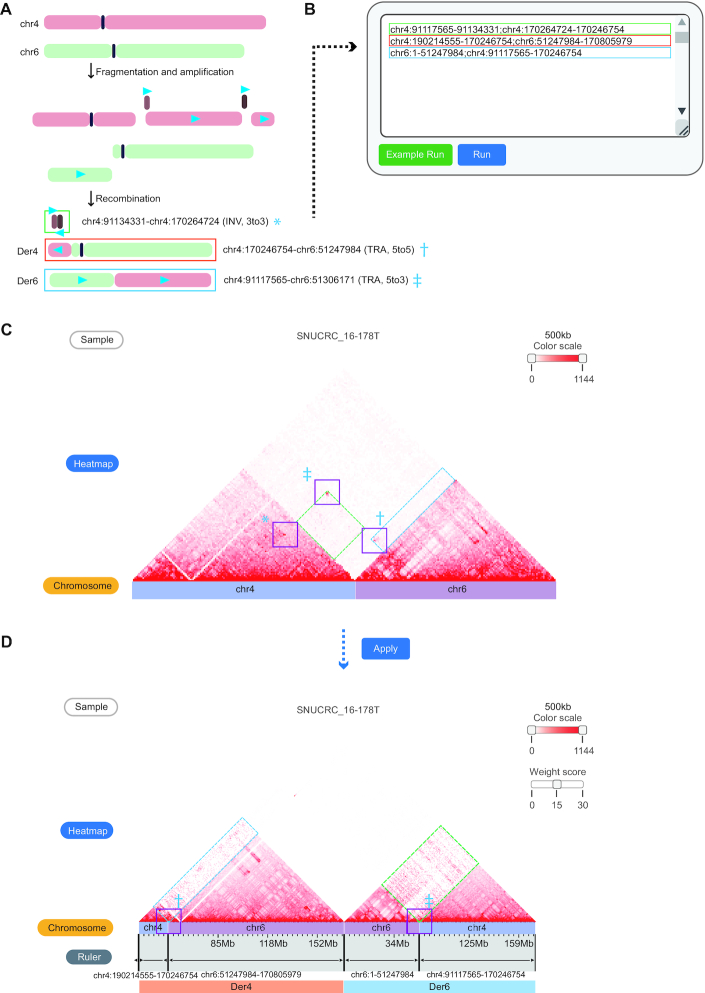
Reorganization of chromatin contacts under user-defined chromosome order. (**A**) Estimated recombination process between chromosomes 4 and 6 of the ‘SNUCRC_16–178T’ tumor sample. The blue triangles indicate the 5′ to 3′ direction of the fragments. Breakpoint coordinates and types of three WGS-identified structural variations are also shown, marked with an asterisk (*), dagger (†), and double dagger (‡). (**B**) A semicolon (;) separates the coordinates to command rearranged genomic fragments. The color of the boxes indicates the formatted text corresponding to each derivative chromosome (Der) shown in (A). (**C**) Original Hi-C contact map (reference genome) of a complex rearrangement sample. Bars below the Hi-C contact map indicate different chromosomes by each color. Purple boxes indicate enriched interactions at the SV breakpoints, which correspond to the coordinates in (A) (*, † and ‡). Dashed line boxes (green and blue) indicate translocation signals between two chromosomes. (**D**) Reorganized Hi-C contacts between chromosomes based on the user-given chromosome order (3′ to 3′ INV fragment in (A) is not visible due to its small fragment size). Translocation signals (dashed line boxes in C) and interactions enriched at the breakpoints (purple line boxes with † and ‡) were fitted to derivative chromosomes after the rearrangement of the Hi-C contact map. Translocation signals can be enhanced by a user-defined parameter (Weight score) for better visualization.

For example, a complex rearrangement event between chromosomes 4 and 6 in the ‘SNUCRC_16–178T’ tumor sample (stage III colorectal cancer) is shown in Figure 4. The estimated recombination processes, including amplification of small fragments (brown and dark brown fragments) and translocations between two chromosomes, are shown in Figure 4A. WGS-based representation of these SVs simply shows the list of breakpoints, limiting the intuitive interpretation of rearranged chromosomes in the 3D nuclear space. To resolve this issue, the third module takes user-defined genomic coordinates and generates a rearranged Hi-C contact map. As an input of the module, the order of the rearranged chromosomes is uploaded with a semicolon (‘;’) delimiter, in which areas that are not entered are treated as genomic deletions (Figure 4B). Running the module generates a Hi-C contact map of involved chromosomes based on the reference genome (Figure 4C) and a reorganized Hi-C contact map based on the given chromosome order (Figure 4D). The recombination result forms smooth triangular shapes that resemble normal Hi-C contact maps, thus indicating the formation of putative TAD fusion or neo-TADs between the rearranged chromosome regions.

## DISCUSSION

The usefulness of Hi-C data in the interpretation of noncoding structural variations has recently been highlighted, revealing the regulatory effects of complex genomic rearrangements in cancer. We updated the 3DIV to provide vast amounts of 3D cancer genome data that cover multiple cancer-types with visualization tools that are expected to be widely used in this field.

As the Hi-C protocol and ‘C’-technologies continue to evolve with the production of various datasets (26,30,31), the 3DIV database will continue to expand in the future to support more experimental results with unique visualization tools.

Despite the advantages of the 3DIV in exploring the higher-order chromatin structure of various samples, there are several limitations to be resolved. The current version of the 3DIV has focused on large-scale structural variations as they greatly impact on the 3D cancer genome; thus, we could not fully address the effect of all types of genetic variations such as copy number alterations. In addition, the incompatibility between 3DIV and the most popular and user-friendly visualization tools such as Juicebox (32) and HiGlass (33) limits the scalability of 3DIV. The functions for the Hi-C contact map manipulation according to the structural variations were not supported by Juicebox and HiGlass, thus independent matrix format data structures were developed and applied to the current version of 3DIV despite the compatibility issues. The discussed limitations will be resolved in future updates.

In summary, the 3DIV database was updated with 3D cancer genome data based on most of the publicly available Hi-C data with powerful visualization and manipulation functions, which are expected to be highly useful in understanding the complexity of cancer biology.

## Supplementary Material

gkaa1078_Supplemental_Files
