# Supplementary material for: 3DIV update for 2021: a comprehensive resource of 3D genome and 3D cancer genome
Source: Nucleic Acids Res. 2020 Nov 27;49(D1):D38–46. doi: 10.1093/nar/gkaa1078 (PMC7778885; doi:10.1093/nar/gkaa1078)

Kim et al Supplementary Figure 1

A

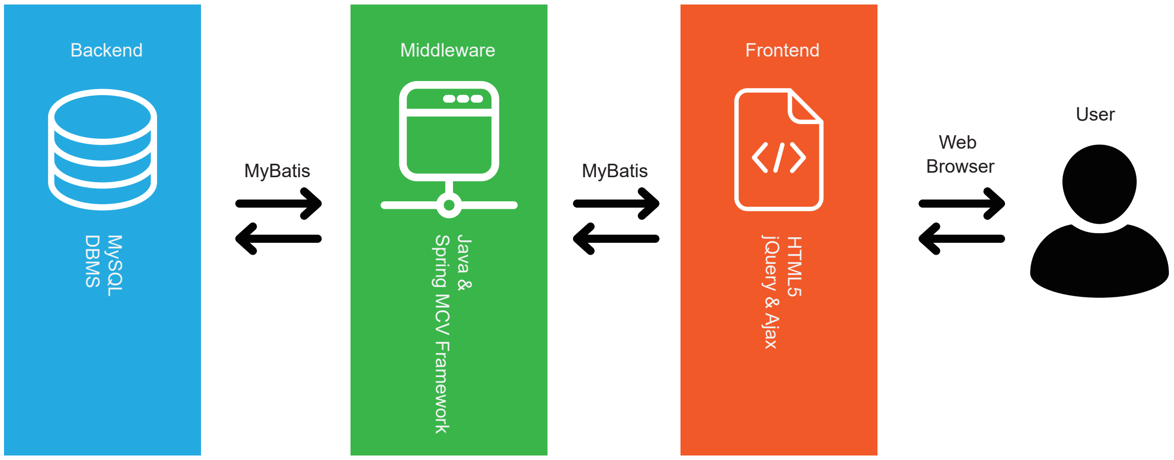

Kim et al Supplementary Figure 2

A

☐	Sample	Bait
☐	GM	chr21:28217728
☐	H1	chr21:28217728
☐	HCmerge	chr21:28217728
☐	IMR90	chr21:28217728
☐	LV	chr21:28217728

Example Run Run

B

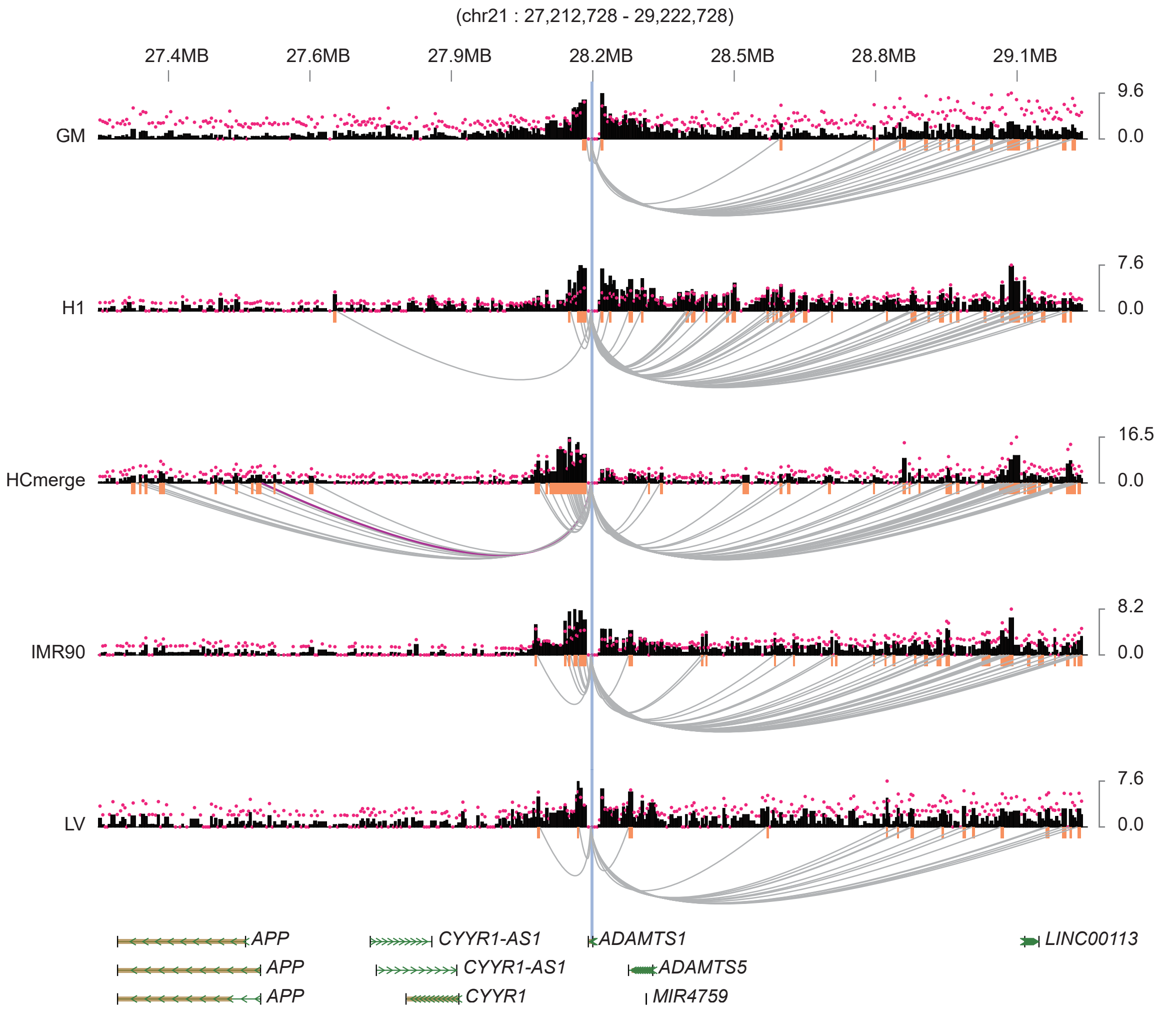

Supplement: gkaa1078_Supplemental_Files [file gkaa1078_supplemental_files.zip › Kim_et_al_SupplFig_merged.pdf]
