# Supplementary material for: 3DIV update for 2021: a comprehensive resource of 3D genome and 3D cancer genome
Source: Nucleic Acids Res. 2020 Nov 27;49(D1):D38–46. doi: 10.1093/nar/gkaa1078 (PMC7778885; doi:10.1093/nar/gkaa1078)
Supplement: gkaa1078_Supplemental_Files [file gkaa1078_supplemental_files.zip › Kim_et_al_Supplmethod_Rev2.pdf]

Supplementary Methods for Kim K et al.

3DIV update for 2021: a comprehensive resource of 3D genome and 3D cancer genome

Collection and processing of Hi-C sequencing data

For the updates, we examined all Hi-C data published to the Gene Expression Omnibus (GEO) after July 2017 and in-house generated 52 cancer Hi-C data. FASTQ format files were downloaded from the Short Read Archive (SRA) using the SRA Toolkit. FASTQ files of replicates were merged and considered as a single sample. Using BWA-MEM (1) with –M option, FASTQ files were aligned to the human reference genome 38 (hg38). The generated SAM files from each FASTQ file were merged into a paired-end BAM file after removing chimeric reads and low-quality reads (MAPQ < 10). Putative self-ligation reads were removed by eliminating reads with mate distances shorter than 15 kb. For 'cancer Hi-C' samples, both *cis* and *trans* reads were used. The filtered BAM file was processed by Picard to remove PCR duplicates. In the case of samples for expanding preexisting normal Hi-C samples in the original 3DIV database, inter-chromosomal interactions were filtered as we focused on the *cis* interactions only. Coverage profiles of chromosome 1 to X at resolutions of 5 kb and 40 kb were obtained by using 'coverageBed' from BEDTools (2). Samples with too low sequencing depth (< 10 M valid reads for tumor samples and < 5 M valid *cis* reads for normal samples) were excluded from the downstream analysis procedure.

For promoter-capture Hi-C data, we used the previously processed results (3), thus providing coordinates of the hg19 reference genome.

Normalization, scaling, and downstream analysis of Hi-C data

To remove experimental biases that are innate in raw Hi-C data, a negative binomial model-based implicit normalization method named 'covNorm' (https://github.com/kaistcbfg/covNormRpkg/) was applied (3,4). In covNorm, all biases were assumed to appear in the form of ligated read coverage for a given genomic region; thus, biases can be normalized by estimating and normalizing the coverage-dependent signal strength. After that, covNorm also performs distance-dependent signal normalization as well as computing significant interactions. During a negative binomial regression, zero interactions were removed in *cis* Hi-C contact maps, and interactions larger than 1 were considered in *trans* Hi-C contact maps, as a large portion of the values close to zero can cause unintended regression results.

In the case of normal Hi-C samples, both coverage-based bias removal and distant dependent background normalization were applied. Only *cis* interactions within a 2 Mb distance were considered. Hi-C contact maps with a resolution of 5 kb were generated, and a 2D convolution function (Gaussian kernel vector length of 7 generated by MATLAB 'gausswin(7)' function) was applied to smooth the highly sparse 5 kb resolution Hi-C contact maps. Coverage normalization followed by distance-dependent background removal was applied to the final 5 kb-resolution Hi-C contact maps. Hi-C contact maps with a 40 kb resolution containing interactions at all distances were also prepared and normalized separately for calling topologically associating domains (TADs). TopDom (5) and DomainCaller (6) were used for TAD calling.

In the case of 'cancer Hi-C' samples, *cis* interactions of each chromosome and concatenated *trans* reads were separately normalized. Only coverage-based normalization was applied, as cancer samples show frequent rearrangements in the genome; thus, the measurement of distance-dependent signals based on the reference genome is not suitable. Both *cis* and *trans* Hi-C contact maps were prepared at a 40 kb and 500 kb resolution.

Each sample has various sources, different sequencing depths, and various library complexities. For proper comparisons between multiple samples, a scale adjustment process was applied to fit interaction frequency values. The mean and standard deviation of normalized chromatin contacts from all samples were obtained, and the average of all means/standard deviations were computed. By scaling each sample's mean and standard deviation to be the

same as the average of all samples' means/standard deviations, we removed the scale bias in Hi-C contact maps to enable comparison between two different samples.

ChIP-seq data processing and super-enhancer annotation

To provide chromatin signatures of normal Hi-C samples, 310 ChIP-seq samples were downloaded and processed (Supplementary Table S2). The BWA-MEM with –M option was used to map reads from FASTQ files to the hg38 reference genome, followed by removing PCR duplicates with Picard and filtering low-quality reads (MAPQ <10). The signal enrichment score per 5 kb bin was computed by selecting the maximum 100 bp resolution input normalized RPKM value within the bin. Super-enhancer information was obtained from dbSUPER (7). The ChIP-seq results were provided with the matched Hi-C sample, if available. If the ChIP-seq results were not available, the most similar ChIP-seq results based on ENCODE were annotated and provided.

GWAS catalog data processing

The GWAS catalog (8) was used to provide disease-associated single nucleotide polymorphism (SNP) information in the database. Catalog version 1.0-e96-r_r2019-07-30 based on the hg38 reference genome was used. The SNPs located at chromosome 1 to X were parsed and used.

Structural variation data processing

The list of structural variations reported in the given browsing genomic regions is provided for the Cancer Hi-C samples. In the case of samples with matched WGS data, somatic SV candidates were called based on Delly software (9) and filtered by examining read tags and supporting split reads according to a previously published protocol (10).

For samples without available matched WGS data, published SVs were downloaded from the International Cancer Genome Consortium (ICGC), which includes data from The Cancer Genome Atlas (TCGA) (11) and the Pan-Cancer Analysis of Whole Genomes (PCAWG) consortium (12). For each cancer type, a list of common large SVs was obtained by selecting SVs that had breakpoint sizes larger than 1 Mb and reported at least two samples. Large-sized SVs were selected as over 1Mb genomic rearrangements are required to be visible on Hi-C contact maps (13).

Reference

1. Li, H. and Durbin, R. (2010) Fast and accurate long-read alignment with Burrows-Wheeler transform. *Bioinformatics*, **26**, 589-595.
2. Quinlan, A.R. and Hall, I.M. (2010) BEDTools: a flexible suite of utilities for comparing genomic features. *Bioinformatics*, **26**, 841-842.
3. Jung, I., Schmitt, A., Diao, Y., Lee, A.J., Liu, T., Yang, D., Tan, C., Eom, J., Chan, M., Chee, S. *et al.* (2019) A compendium of promoter-centered long-range chromatin interactions in the human genome. *Nat Genet*.
4. Yang, D., Jang, I., Choi, J., Kim, M.S., Lee, A.J., Kim, H., Eom, J., Kim, D., Jung, I. and Lee, B. (2018) 3DIV: A 3D-genome Interaction Viewer and database. *Nucleic Acids Res*, **46**, D52-D57.
5. Shin, H., Shi, Y., Dai, C., Tjong, H., Gong, K., Alber, F. and Zhou, X.J. (2016) TopDom: an efficient and deterministic method for identifying topological domains in genomes. *Nucleic Acids Res*, **44**, e70.
6. Dixon, J.R., Selvaraj, S., Yue, F., Kim, A., Li, Y., Shen, Y., Hu, M., Liu, J.S. and Ren, B. (2012) Topological domains in mammalian genomes identified by analysis of chromatin interactions. *Nature*, **485**, 376-380.

7. Khan, A. and Zhang, X. (2016) dbSUPER: a database of super-enhancers in mouse and human genome. *Nucleic Acids Res*, **44**, D164-171.
8. Buniello, A., MacArthur, J.A.L., Cerezo, M., Harris, L.W., Hayhurst, J., Malangone, C., McMahon, A., Morales, J., Mountjoy, E., Sollis, E. *et al.* (2019) The NHGRI-EBI GWAS Catalog of published genome-wide association studies, targeted arrays and summary statistics 2019. *Nucleic Acids Res*, **47**, D1005-D1012.
9. Rausch, T., Zichner, T., Schlattl, A., Stutz, A.M., Benes, V. and Korbel, J.O. (2012) DELLY: structural variant discovery by integrated paired-end and split-read analysis. *Bioinformatics*, **28**, i333-i339.
10. Lee, J.J., Park, S., Park, H., Kim, S., Lee, J., Lee, J., Youk, J., Yi, K., An, Y., Park, I.K. *et al.* (2019) Tracing Oncogene Rearrangements in the Mutational History of Lung Adenocarcinoma. *Cell*, **177**, 1842-1857 e1821.
11. Cancer Genome Atlas Research, N., Weinstein, J.N., Collisson, E.A., Mills, G.B., Shaw, K.R., Ozenberger, B.A., Ellrott, K., Shmulevich, I., Sander, C. and Stuart, J.M. (2013) The Cancer Genome Atlas Pan-Cancer analysis project. *Nat Genet*, **45**, 1113-1120.
12. Consortium, I.T.P.-C.A.o.W.G. (2020) Pan-cancer analysis of whole genomes. *Nature*, **578**, 82-93.
13. Dixon, J.R., Xu, J., Dileep, V., Zhan, Y., Song, F., Le, V.T., Yardimci, G.G., Chakraborty, A., Bann, D.V., Wang, Y. *et al.* (2018) Integrative detection and analysis of structural variation in cancer genomes. *Nat Genet*, **50**, 1388-1398.

Supplementary Table and Figure Legends for Kim K et al,

3DIV update for 2021: a comprehensive resource of 3D genome and 3D cancer genome

Table S1. Summary of processed samples and sequencing read information. Note: Samples marked with 'QC not passed' were processed, but the quality of the data was too low.

Table S2. Summary of processed ChIP-seq data.

Figure S1. The three-layered architecture of the updated 3DIV.

Figure S2. Example of the capture Hi-C visualization function. (A) The input of the module shows the selected samples (GM: GM12878 + GM19240 lymphoblastoid cell line, H1: H1 embryonic stem cell, HCmerge: hippocampus, IMR90: Fibroblast cells, and LV: left ventricle) and the given bait locus. (B) Promoter-centered chromatin interactions of the given samples for the *ADAMTS1* gene. Blue vertical bar indicates bait loci; black bars and red dots indicate normalized interaction frequencies over expected values, and arcs (purple: promoter-promoter interaction and gray: promoter-other interaction) with orange boxes indicate significant long-range interactions (-log10 *p*-value > 2).
